# Sentinel node biopsy in early breast cancer at the Hospital Comarcal La Linea (Spain)

**DOI:** 10.3332/ecancer.2013.353

**Published:** 2013-09-23

**Authors:** Jack Antonio Díaz Brito, Sofía Vásquez Navarrete, Juan Antonio Muñoz, Yolanda Santaella Guardiola, José González Sánchez, Vicente Vega Ruiz, Miguel Velasco García

**Affiliations:** 1 General Surgery Department, Hospital La Línea, La Línea, Cádiz, Spain; 2 Pathology Department, Hospital La Línea, La Línea, Cádiz, Spain; 3 Radiology Department, Hospital La Línea, La Línea, Cádiz, Spain; 4 Nuclear Medicine Department, Hospital Punta Europa, Algeciras, Cádiz, Spain; 5 General Surgery Department, Hospital Universitario Puerto Real, Puerto Real, Cádiz, Spain

**Keywords:** early breast cancer, axillary staging, sentinel node biopsy, SNOLL, micrometastasis

## Abstract

**Objective:**

Our objective was to determine the identification and the percentage of false negatives in sentinel node biopsies in patients with early breast cancer at the Hospital La Línea (Spain), during the period between November 2007 and September 2010.

**Methods:**

We collected 50 patients with early breast cancer, without clinical and ultrasonographic involvement of axillary nodes, from November 2007 to September 2010. We used the vital dye in the first 20 patients and the combined technique of vital dye and albumin labelled with technetium 99 in the other 30 patients. The site of injection for patients using blue dye was subdermal for palpable tumours and periareolar for non-palpable tumours. The technique of injection with the radioisotope for patients for palpable and most non-palpable tumours was the periareolar technique. We used albumin labelled with technetium 99. In seven patients with non-palpable tumours, we used the sentinel node occult lesion localisation (SNOLL) technique. The sentinel node biopsy was examined during surgery, with the frozen section examination and imprint as follows: the sentinel node was cut in three transversal sections along the axis and five frozen sections of each portion were done at a distance of 60 μm each; in total, 15–20 frozen sections and three imprints were done for each sentinel node. The axillary dissection was completed in the first 17 patients, and we performed total axillary dissection on the remaining patients if the sentinel node was positive for metastasis.

**Results:**

The sentinel nodes were identified in 49 of 50 patients (98%). The patient in whom we did not identify the sentinel node was a patient in the combined technique. The number of nodes identified in the patients with vital dye was one sentinel node, and with the combined technique, it was two sentinel nodes. The false-negative rate was 8% (four patients); the micrometastasis was the principal factor of the false-negative rate (*p *< 0.05). The cases of false negatives were present at the beginning of the study with the use of the blue dyes; this factor was statistically significant (*p *< 0.05). The tumour size, the vascular invasion, and the periganglionar adipose tissue invasion were statistically significant for the presentation of axillary metastasis (*p *< 0.05).

**Conclusion:**

This study shows that the micrometastasis and the use of vital dye were the principal factors for the presentation of the false-negative rate. The size of the tumour, the vascular invasion, and the periganglionar adipose tissue invasion were statistically significant for the appearance of the axillary metastasis.

## Introduction

Cancer is the second leading cause of death in developed countries after cardiovascular and cerebrovascular diseases [1].

Breast cancer is the most common malignancy among women and is the leading cause of cancer deaths in women [2]. In Spain, the frequency increases by 2% annually. The incidence is still increasing every day since 1950. This situation can be explained by the increase of elderly women, detection using mammography screening, dietary changes, and the delay in having children [3, 4].

Axillary involvement is the most important predictor in breast cancer. This fact determines the importance of axillary staging by sentinel node biopsy. Recent studies have shown that minimal involvement in sentinel node biopsy (isolated tumour cells, micrometastasis) and even macrometastasis carries the same overall survival and disease-free survival to 5 years, performing sentinel node biopsy or axillary dissection [5–9].

In 2007, we performed the first sentinel node biopsy for breast cancer in the Campo de Gibraltar. Since then, we have treated 50 women with early breast cancer, to realise this technique and to avoid the morbidity associated with axillary dissection.

## Materials and method

### Patients

We collected patients who attended the outpatient clinic of the Hospital La Línea, referred by their medical practitioners and from the screening program for the surveillance of breast cancer. Our Hospital has 180 beds and covers a population of 130,000 inhabitants.

### Inclusion criteria

- We admit patients with early breast cancer tumours T1–T2 without clinical and/or ultrasonographic axillary involvement.
- Patients with multifocal or multicentric early breast cancer.
- Patients with ductal carcinoma *in situ* in the breast that requires total mastectomy.
- Patients with previous surgery of the breast for benign conditions.

### Exclusion criteria

- Patients with clinical or ultrasonographic involvement of axillary lymph nodes.
- Patients with advanced breast cancer and in treatment with neoadjuvant chemotherapy.
- Pregnant patients.
- Inflammatory breast cancer patients.

### Nuclear medicine

The patient is hospitalised the day after surgery. All the patients are transferred by ambulance to the Nuclear Medicine Service of the Hospital Punta Europa Algeciras. The distance is 18 km. The Nuclear Medicine Services use a nanocoll of human serum albumin labelled with 99Tc, size 80–90 nm.

**Figure 1.**
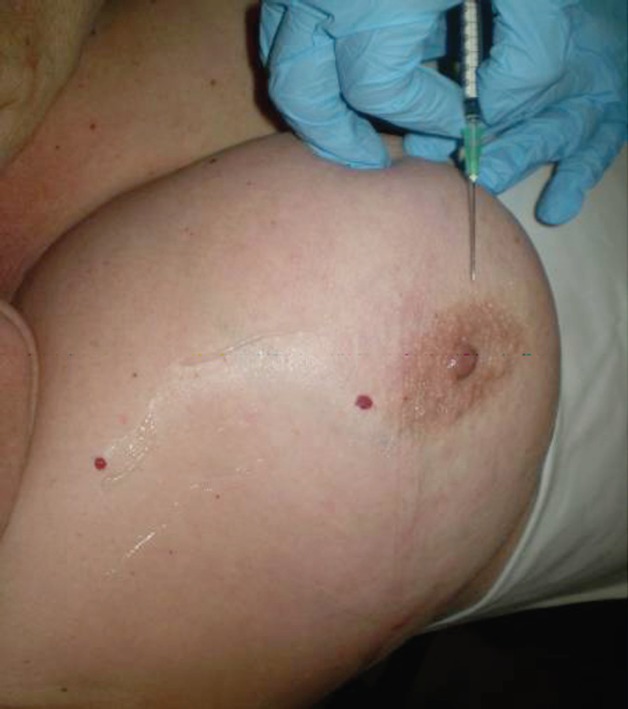
Radioisotope injection, palpable tumours.

Previous asepsis of the breast, 4 millicurie dissolved in 0.1–0.2 mL of normal saline are injected in the periareolar area for the patients with palpable tumours (Figure 1).

In most patients with non-palpable tumours, we use the injection in the periareolar area as explained before. In seven patients, we used the SNOLL technique, which is explained below.

The SNOLL technique is sentinel node occult lesion localisation, which identifies the sentinel node and the non-palpable lesion of the breast. This technique uses a nanocoll of human serum albumin, which is 80 nm in size and is then injected into the tumour using ultrasonographic guidance. After the injection, a gammagraphic image will be done to show the sentinel node and the tumour. On the day of surgery, the nuclear medicine doctor and the general surgeon identify the tumour and the sentinel node, with the help of the gamma probe [10, 11].

**Figure 2.**
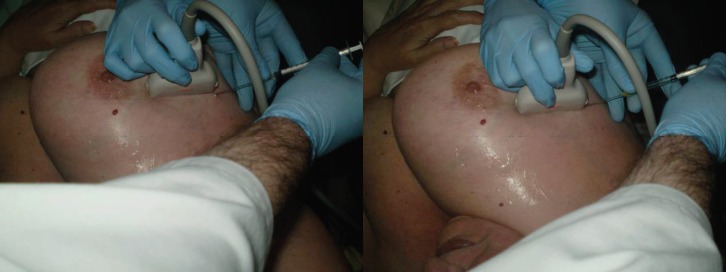
SNOLL technique, ultrasonographic guidance.

The Nuclear Medicine Service of the Hospital Punta Europa (Algeciras) injected 4 millicurie of nanocoll of human serum albumin dissolved in 0.2–0.3 mL of normal saline into the tumour, for the SNOLL technique and with ultrasonographic guidance (Figure 2).

The gammagraphic images are made at 12 minutes, 30 minutes, and 1 hour after the injection of the radioisotope, until the sentinel and the tumour were displayed. The day after, in the operation room, the nuclear medicine doctor and the general surgeon identify the tumour and sentinel node with the help of the gamma probe.

### Blue dye

**Figure 3.**
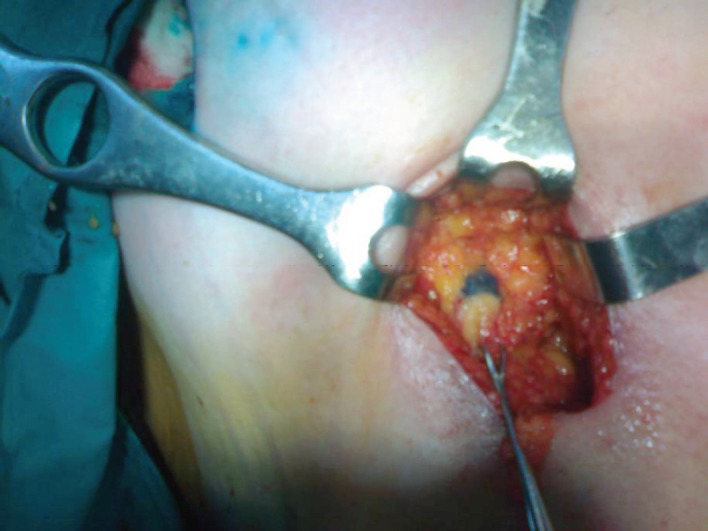
Sentinel node biopsy identification with blue dye.

We used methylene blue in 48 patients and isosulfan blue in two patients. The site of injection for the palpable tumours was the subdermal area near to the tumour, and in the patients with non-palpable tumours, it was the periareolar area. The blue dye was injected immediately after the anaesthesia induction, in a percentage of 3 mL (Figure 3).

### Sentinel node frozen section

**Figure 4.**
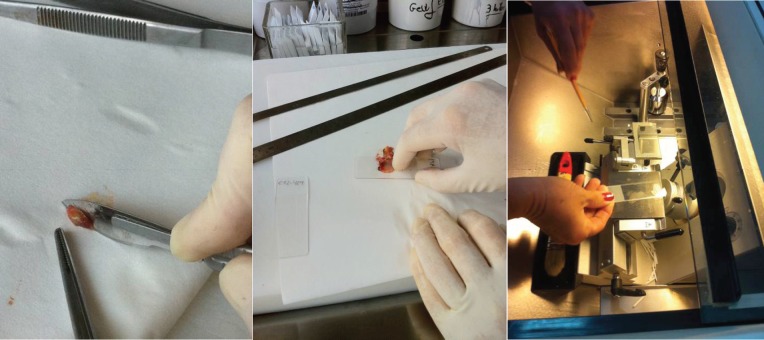
Imprint and frozen section examination of the sentinel node.

When the sentinel node was identified, by blue staining or by the combined technique, it was sent to the pathology department for intraoperative examination. The sentinel node was cut into three portions across the long axis. The pathologist realised three imprints of these cuts, and then each portion of the sentinel node was cut by frozen section at a distance of 60 μm each. In total, three imprints and 15–20 frozen sections were done for each sentinel node. The remaining tissue of the sentinel node was left to be examined with paraffin (Figure 4).

### Statistical analysis

A statistical analysis was performed with the statistical support of Cadiz University using Windows/SPSS 20.0. We described the next statistical techniques: frequency tables for qualitative variables, descriptive tables for quantitative variables, Pearson coefficient correlation, analysis of variance (ANOVA), and chi square test. In all cases, statistical t test was done with a significance level *p *< 0.05.

## Results

During the period from November 2007 to September 2010, we analysed 50 patients, all women who completed the criteria for sentinel node biopsy for early breast cancer. The average age of the patients was 60 years (range: 29–84 years).

The tumour characteristics are displayed in Table 1.

Infiltrating ductal carcinoma, middle grade, and positive hormonal receptors were the principal tumour characteristics in our study.

### Sentinel node identification

We used the blue dye in all of the patients. In the first 20 patients, we used the vital dye for the identification of the sentinel node, and in the other 30 patients, we used the combined technique.

The rate of identification of the group of vital dye was 100%, and with the combined technique, it was 96%. Complete identification of 50 patients was 98% (Table 2).

There was a patient in whom we did not identify the sentinel node; this patient was a 66-year-old woman with a tumour of 2 cm in size in the upper inner quadrant of the left breast, an infiltrating ductal carcinoma with middle grade. We did an axillary dissection, and one of the 11 lymph nodes was compromised by the tumour.

### False negative

In our study, we found an 8% (four patients) false-negative rate. There were three patients with micrometastasis and another one with macrometastasis who were not seen in the frozen section examination but were seen in the paraffin examination.

The false negatives were presented at the beginning of the study with the use of blue dyes. The chi square test, the fisher test, and the likelihood ratio showed a statistically significant difference between the use of blue dyes and the presentation of false negatives (*p* < 0.05).

The presence of micrometastasis was statistically significant for the presentation of the false negatives (*p* < 0.05).

In our study, due to administrative and technical problems, we did not use immunohistochemical staining in the frozen section examination of the sentinel node.

### Axillary metastasis

The correlation between the qualitative variables and the quantitative variables was performed through ANOVA. In our study, we found that the size of tumour, the vascular invasion, and the periganglionar adipose tissue invasion were statistically significant for the presence of axillary metastasis (*p* < 0.05).

## Discussion

Breast cancer is the most common cancer among women and is the primary cause of death for cancer in this population. The literature review showed that the most common tumour characteristics are size between 1 and 2 cm, infiltrating ductal carcinoma represents 71.4%–89.3%, the most common grade is the middle grade, vascular invasion ranging from 31.6% to 42.5%, oestrogen receptors up to 81%, and progesterone receptors up to 64% [12–15].

In our study, the tumour characteristics are similar to the literature; the mean tumour size was 1.9 cm; the infiltrating ductal carcinoma represents the 72%; the middle grade represented 64% of cases; and the oestrogen receptors and progesterone receptors were 78 and 82%, respectively.

The percentage of identification of the sentinel node with blue dyes in the large series ranging between 65% and 97% [16–19], and with the combined technique, it was between 96% and 99.7% [20–24]. Our study shows that sentinel node identification with the use of blue dyes was 100%, and with the combined technique, it was 96.6%, overall 98%.

In Table 3, we compare our study with other reports in the literature concerning the percentage of identification rate of false negative and other parameters.

The rate of false negative in the literature varies between 0% and 29%, average 8%. This great disparity is due to the number of patients, inclusion criteria, detection rate, and the technique with blue dyes or in combination with radioisotopes [27].

This study shows that the false-negative rate was 8%; micrometastasis was the principal factor for the presentation of these cases of false negatives (*p* < 0.05).

The use of blue dyes in our study was statistically significant for the presentation of false negatives (*p *< 0.05).

The size of tumour, the vascular invasion, and the periganglionar adipose tissue invasion are independent and combined factors for the presentation of axillary metastasis for breast cancer [28–29]. In this study, there was a statistical significance between the presentation of axillary metastasis and the size of tumour and the presence of vascular invasion and the periganglionar adipose tissue invasion (*p* < 0.05).

## Conclusion

This study shows that a rate of sentinel node identification using the blue dyes was 100%, and with the combined technique, it was 96.6%, overall 98%. There was a statistical significance with the presentation of the false-negative cases and the use of blue dyes for the sentinel node identification, because all the false-negative cases were present at the beginning of the study, with the use of this method of identification. Micrometastases were the principal factor for the presentation of the false-negative cases, which were not seen at the frozen section examination of the sentinel node. The size of tumour, the vascular invasion, and the periganglionar adipose tissue were statistically significant for the presentation of axillary lymph node metastasis.

## Figures and Tables

**Table 1.** Tumour characteristics.

Tumour characteristic present	Number present of 50 cases	Percentage (%)
Infiltrating ductal carcinoma	36	72
Grade II	32	64
Intraductal component extension	42	84
Necrosis	11	22
Vascular invasion	15	30
Oestrogen receptor	39	78
Progesterone receptor	41	82
Her2-neu	14	28
Ki-67^a^	25	50

a The cut-off for the Ki67 was 10%.

**Table 2.** Sentinel node identification.

Technique	Patients	Identification	NI	Percentage
Blue dye	20	20	0	100%
Combined	30	29	1	96.6%
Total	50	49	1	98%

NI: Not identified.

**Table 3.** Studies of sentinel node identification and other parameters.

Study	No. Ptes	FN (%)	Rad.	VD	ID Rad.	ID VD	Total ID
Krag [25]	22	1	Sulfur colloid 0.4 mc	–	81%	–	81%
Giuliano [16]	172	2.8		Lyph 3–5 mL	–	65%	65%
Olson [22]	223	5	Sulfur colloid 0.4 mc	Lyph 4 mL	91%	91%	97%
Canavese [24]	212	6.5%	Human albumin 5–10 MBq	Patent Blue-V 5 mL	94.1%	73.8%	98.7%
Veronesi [26]	376	6.7%	Human albumin 5–10 MBq	–	98.7%	–	98.7%
Diaz B^a^	50	8%	Human albumin 4 mc	Met blue 3 mL Lyph 3 mL	96.6%	100%	98%

No. Ptes: Number of Patients; FN: false-negative rate; Rad: radioisotope; VD: vital dye; ID: identification; mc: millicurie; mL: millilitre; ND: not determined; μci: microcurie; Lyph: lymphazurin; Met: methylene.

a Our study.
